# Application of Plant-Derived Nanoparticles (PDNP) in Food-Producing Animals as a Bio-Control Agent against Antimicrobial-Resistant Pathogens

**DOI:** 10.3390/biomedicines10102426

**Published:** 2022-09-28

**Authors:** Daniel Jesuwenu Ajose, Tesleem Olatunde Abolarinwa, Bukola Opeyemi Oluwarinde, Peter Kotsoana Montso, Omolola Esther Fayemi, Adeyemi Oladapo Aremu, Collins Njie Ateba

**Affiliations:** 1 Food Security and Safety Focus Area, Faculty of Natural and Agricultural Sciences, North-West University, Private Bag X2046, Mmabatho 2735, South Africa; 2 Antimicrobial Resistance and Phage Bio-Control Research Laboratory, Department of Microbiology, Faculty of Natural and Agricultural Sciences, North-West University, Private Bag X2046, Mmabatho 2735, South Africa; 3 Department of Chemistry, Faculty of Natural and Agricultural Sciences, North-West University, Private Bag X2046, Mmabatho 2735, South Africa; 4 Indigenous Knowledge Systems (IKS) Centre, Faculty of Natural and Agricultural Sciences, North-West University, Private Bag X2046, Mmabatho 2735, South Africa

**Keywords:** alternative therapy, antibiotics, antimicrobial resistance, foodborne pathogens, green synthesis, multidrug-resistant bacteria, One Health Concept, phyto-nanomedicine

## Abstract

Antibiotics are regularly used in animal husbandry to treat diseases. This practice is beneficial to animals’ health and helps ensure food security. However, the misuse of antibiotics, especially in food-producing animals, has resulted in the advent of antimicrobial resistance (AMR) and its dissemination among foodborne pathogens. The occurrence of AMR in bacteria pathogens that cause infections in animals and those associated with food spoilage is now considered a global health concern affecting humans, animals and the environment. The search for alternative antimicrobial agents has kindled the interest of many researchers. Among the alternatives, using plant-derived nanoparticles (PDNPs) for treating microbial dysfunctions in food-producing animals has gained significant attention. In traditional medicine, plant extracts are considered as safe, efficient and natural antibacterial agents for various animal diseases. Given the complexity of the AMR and concerns about issues at the interface of human health, animal health and the environment, it is important to emphasize the role of a One Health approach in addressing this problem. This review examines the potential of PDNPs as bio-control agents in food-producing animals, intending to provide consumers with microbiologically safe food while ensuring food safety and security, better health for animals and humans and a safe environment.

## 1. Introduction

Milk and meat are some of the most important products derived from animal husbandry. Thus, it is highly pertinent to improve the well-being of food-producing animals, such as cows, goats, ducks, fish and fowl for quality milk, meat and egg production, respectively. Breaches in the various guidelines relating to animal welfare may lead to several diseases, and hence, a decline in animal products [1]. Consequently, milk and meat may become inedible and therefore be discarded, resulting in wastage, failure to meet consumer demands and a decline in the supply of meat, milk, their derivatives and other related products. Other losses associated with animal husbandry are the high cost of treating infected animals, the premature culling of diseased animals and the spread of infections to other animals and humans (zoonotic transmission) which all contribute significantly to economic losses [2]. In addition, microbial products, toxins and enzymes produced by pathogenic bacteria species such as *S. aureus* that are present in milk can cause food-related diseases in humans. More so, the remnants of *S. aureus* in cells can become a source of recurrent infections [3].

Food-producing animals are the main reservoirs for most foodborne pathogens, such as the *Campylobacter* species, the *Salmonella enterica* non-Typhi serotypes, the shiga toxin-producing *Escherichia coli*, *S. aureus*, *Bacillus cereus*, *Listeria monocytogenes* and *Clostridium botulinum* [4,5,6,7,8]. Moreover, foodborne pathogens may arise from different sources which include the environment (water from various sources, animal dung disposal sites and wildlife), as well as human-related animal handling (slaughtering and processing practices, and storage procedures) [9,10]. The ability of these pathogens to produce toxins that cause illness or even death in both humans and animals amplifies their public health significance. Infections caused by these foodborne pathogens are usually treated with standard antibiotics. However, the indiscriminate use of these antibiotics in animals may exert pressure on the environment, and in response, the disease-causing microorganisms may develop resistant mechanisms against the antibiotics [11].

**Figure 1:**
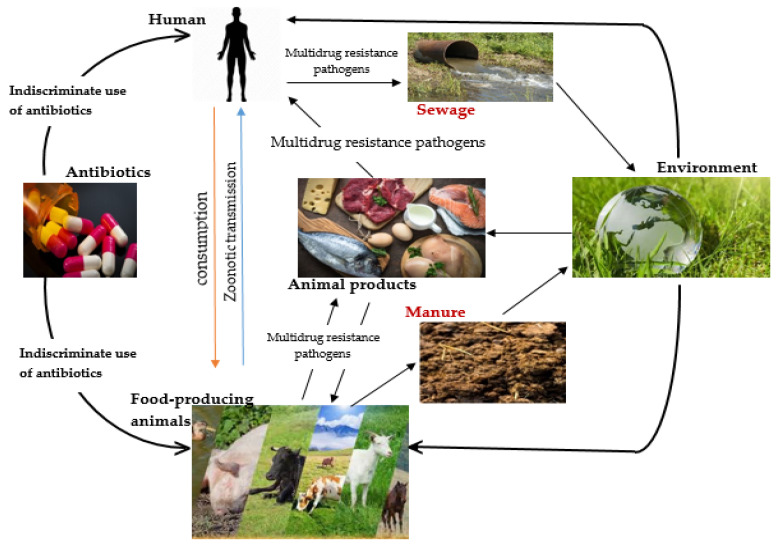
Transmission pathways of antimicrobial resistance between food-producing animals, the environment and humans.

There are probable reservoirs of resistance wherever antibiotics are used, including in humans and animals, on farms, as well as in environments, such as hospitals, water sources, soil, wildlife, and many other ecological niches. This resistance may also be attributed to pollution from sewage, pharmaceutical and industrial waste, and runoff from farm manure (Figure 1) [12]. Bacteria and their genetic materials (DNA and/or plasmids) may readily be transmitted between humans, animals and the environment. AMR is therefore a menace and is defined by complex interactions between distinct microbial populations that influence human, animal and environmental health [13,14]. Hence, actions taken (or not) to combat AMR in one industry may have an impact on other industries [15]. It is thus imperative to combat this global challenge by utilizing coordinated cross-sectoral strategies, such as One Health, that take into consideration the complexity and ecological nature of the problem.

Nanotechnology refers to the system of synthesizing materials of different sizes and shapes at the nanoscale (10^−9^ one-billionth of a meter) level by utilizing matter [16]. These particles with significantly reduced sizes (about 1 to 100 nanometres) possess physical and chemical properties that differ drastically from large-scale materials made up of the same component. This technology has since evolved into a diverse field of applied science and technology and is projected to have an impact on practically every aspect of daily living. Over the last decade, research in this field has expanded, and many types of nanoscale materials are now available in different countries [17].

The increasing development of resistance to antimicrobials traditionally used in the management of animal infections has necessitated the upsurge of alternative approaches employing nanoparticles (NPs) and the use of plants and plant products to counteract the global menace of AMR, thus assuring food safety and security [18]. Hence, this review focuses on green synthesized NPs from plant extracts which act as bio-control agents in the management of AMR in foodborne pathogens, with the focus on the One Health approach.

## 2. Emergence of Multidrug Resistance (MDR) Pathogens in the Food Chain

A leading public health issue in recent decades has been the growth of multi-drug resistant (MDR) bacteria. The prevalence of these pathogens in animal-derived products, including milk and meat, has risen considerably and their potential to evolve new features, notably MDR, is significant [19,20]. The upsurge in MDR bacteria has hitherto remained undisclosed to the animal food-service sector because there has previously been virtually no communication of their occurrence in animal-based products. However, more recently, new exceptions, such as mobile colistin-resistant (mcr) strains and New Delhi metallo-β-lactamase-1 (NDM-1)-producing variants in food-producing animals, have been surfacing as discrete pools of colistin and β-lactam resistance, along with the alternative carbapenem antibiotic-resistant strain [21].

The use of antibiotics has long been linked to the emergence of drug resistance [22]. When an antibiotic is ingested, it kills vulnerable bacterial cells, while the resistant ones continue to proliferate and become the dominant strains [12]. This provides opportunities for the transfer of resistant genes to their offspring [12]. Given that the food supply chain is an ecological niche made up of diverse biological points in which significant amounts of drugs are utilized and scores of bacteria coexist, food-producing animals, seafood, meat and milk are regarded as significant pools for the proliferation of antimicrobial-resistant bacteria [23].

Antibiotic resistance may occur in one of two ways. Firstly, it can occur as intrinsic resistance, in that an existing natural composition in the bacterial species provides that specific species with the potential to resist the action of an antibiotic [24]. During their developmental stages, bacterial cells amass genetic flaws in their chromosomes and/or plasmids and pass down the same to their daughter cells through vertical gene transfer (VGT), thus accounting for natural or inherent resistance [24]. The other mechanism, termed “acquired resistance”, involves the transfer of genetic materials between and within bacterial species. This mechanism involves the lateral transfer of the genetic materials in a process called horizontal gene transfer (HGT). These codes are carried on or within selfish genetic elements, including transposons [12].

### Use of Antibiotics in Animal Agriculture, Their Mode of Action and Resistance Mechanisms

Antibiotics are routinely utilized in animal production to support the health and development of the animals. Producers and consumers as a whole gain certain financial advantages from this strategy. For a very long time, antibiotics have been thought of as the first line of defence against bacterial infections in animal husbandry. They are still essential medical drugs that must be handled with caution when treating sick animals, thus ethical livestock production does not have to completely forego their use. Antibiotics can be categorized according to their modes of action, which include the inhibition of cell wall synthesis, the suppression of nucleic acid synthesis, the repression of ribosome function, the inhibition of cell membrane function, and the inhibition of folate metabolism [25] (Table 1).

However, there are certain issues connected to the use of antibiotics in animal agriculture. Given that the antibiotics used are identical to or substitutes for the antibiotics used in human treatment procedures, there has been great worry that repeatedly exposing these animals to low dosages of antibiotics adds considerably to antimicrobial resistance. Livestock alone consumes 50–80% of all antibiotics produced in the majority of the developed countries [26]. Animals are frequently given less antibiotics than are used for therapeutic purposes when using them as a growth promoter. Due to the frequent exposure of bacteria to sub-lethal doses of antibiotics and the favourable conditions for the selection and maintenance of resistance features, this approach is more likely to exert significant pressure on the emergence of antimicrobial resistance mechanisms [27] (Table 1).

**Table 1:** Mode of action and mechanisms of resistance of antibiotics.

Antibiotic Family	Mode of Action	Mechanism of Resistance	Reference
β-lactams β-lactamase inhibitors Fluoroquinolones Macrolides, Lincosamides and Streptogamin (MLS) Aminoglycosides Tetracyclines Sulfonamides (Folate pathway inhibitors)	Cell wall synthesis inhibitors. Binds transpeptidase also known as penicillin binding proteins (PBPs) that help form peptidoglycan Inactivates the enzyme; beta-lactamase Hydrolysis of the beta-lactam ring Binds DNA-gyrase or topoisomerase II and topoisomerase IV; enzymes needed for supercoiling, replication and separation of circular bacterial DNA. Binds the bacterial 50S ribosomal subunits; inhibit protein synthesis Bind to the bacterial 30S ribosomal subunit thus inhibit bacterial protein synthesis Bind reversibly to the 30S ribosomal subunit as such blocks the binding of the aminoacyl-tRNA to the acceptor site on the mRNA-ribosome complex Inhibit the bacterial enzyme dihydropteroate synthetase (DPS) in the folic acid pathway, thereby blocking bacterial nucleic acid synthesis	Beta-lactamase production primarily - *bla* genes, Expression of alternative PBPs Production of extended spectrum beta-lactamases (ESBLs) Target modification, Decreased membrane permeability, Efflux pumps Target site modification, Active drug efflux Target site modification (via the action of 16S rRNA methyltransferases (RMTs)), Enzymatic Drug Modification (adenylation, acetylation and phosphorylation), Efflux systems Efflux systems, Target modification, Inactivating enzymes, Ribosomal protection Excessive bacterial production of dihydrofolate reductase (DHFR), Reduction in the ability of the drug to penetrate the bacterial cell wall, Production of altered forms of the dihydropteroate synthetase (DPS) enzyme with a lower affinity for sulfonamides, Hyperproduction of para-amino benzoic acid (PABA), which overcomes the competitive substitution of the sulfonamides	[25,28,29] [30] [31] [32] [33,34] [35,36]

## 3. Annals of One Health Antimicrobial Resistance

Antibiotic resistance is a growing issue of severe public health concern worldwide and is now regarded as a critical One Health issue. Based on a concise historical record, two accounts of some of the antimicrobial resistance issues that have resulted from the use of the same antibiotic classes in humans and animals, as well as the associated complications with competing interests, are described. The first scenario, which focuses on third-generation cephalosporins, demonstrates One Health concerns with an antibiotic that is primarily used for therapeutic purposes in animals and is also used prophylactically in some key conditions. The second scenario is colistin, an older type of antibacterial agent that has long been utilized in animals for medicinal, preventive and growth-promotion objectives, but has only lately gained prominence in the human health arena.

### 3.1. Third-Generation Cephalosporins

Broad-spectrum *beta*-lactam antibiotics, known as third-generation cephalosporins, are routinely utilized in humans and animals. Cefotaxime, ceftriaxone and other members of this group are employed to treat a wide range of infections in humans, including urinary tract, abdominal, lung, and bloodstream infections caused by *E. coli*, *Klebsiella pneumoniae*, and other bacteria, as well as infections caused by *Neisseria gonorrhoeae* [37]. This class of antibiotics has been designated as “critically essential” for human health because of its critical role in the treatment of numerous bacterial infections, in which resistance has become a serious issue [37].

Extended-spectrum *beta*-lactamases (ESBLs) and AmpC *beta*-lactamases are responsible for resistance to third-generation cephalosporins. ESBL genes are easily spread by plasmids, transposons and other genetic elements [38]. Originally thought to be chromosomally associated, AmpC *beta*-lactamases have also been found on plasmids and demonstrated to have been propagated through horizontal transfers throughout Enterobacteriaceae [38]. Unfortunately, resistance to third-generation cephalosporins is frequent in *E. coli* and *K. pneumoniae*, both emanating from serious human infections in many countries [39] and forcing clinicians to rely more heavily on the few remaining antimicrobial classes, such as carbapenems. According to a study by the World Health Organization (WHO) [40], as opposed to susceptibility to infections, patients with third-generation cephalosporin-resistant *E. coli* infections showed a two-fold increase in all-cause deaths, bacterium-attributable mortality and 30-day mortality. *Salmonella* species have also been found to harbour resistance, which is mediated mostly by the CMY-2 AmpC *beta*-lactamase genes that are usually remotely-hosted with genes encoding resistance to other antimicrobial classes, such as tetracyclines, aminoglycosides and sulfonamides [41].

Although much of the proliferation of *E. coli* with ESBL and other *β*-lactamases is assumed to be clonal, the relevant genes have been found in a range of bacteria from humans, animals and the environment [42]. From a One Health perspective, third-generation cephalosporins are favourably considered to be critically essential for both human and animal health (Table 2). As a result, third-generation cephalosporins are widely used either as therapeutic or prophylactic agents, which facilitates the spreading of resistance from animals to humans (Table 1). Another family of antibiotics, the fluoroquinolones, has been used in similar approaches and has thus led to resistance to these antimicrobial agents. Following the mass treatment of chicken flocks, resistance to key antimicrobials has evolved among *Campylobacter jejuni* isolates [43].

### 3.2. Colistin

Colistin is an antibiotic that belongs to the family polymyxin, which has been utilized in human and animal care for more than five decades [44]. Polymyxins, which are toxic to the neurons and nephrons of humans, were hitherto primarily used as colistimethate sodium by inhalation in humans for topical applications and in the nursing of cystic fibrosis patients [44]. Colistin is becoming more popular as a last resort for treating multi-drug-resistant Gram-negative infections, such as carbapenem-resistant *Pseudomonas aeruginosa*, *Acinetobacter baumannii*, *K. pneumoniae*, and *E. coli*, primarily in intensive care units in several countries [45]. Most often, colistin is administered orally to herds of pigs, poultry, and in certain circumstances, calves, for its therapeutic or prophylactic benefits in food-producing animals [44,46]. Colistin is also used as a growth promoter in animals in several countries [47]. Owing to technical problems in phenotypic susceptibility testing, compulsory checks for colistin resistance in *Salmonella* and *E. coli* from animals and some food products began in Europe as recently as 2014 [44,45,46].

A study reported that among the 162 colistin-resistant *E. coli* isolates from chicken, MDR was found in 91.4% of the cases [45]. In the recent past, acquired colistin resistance was assumed to be limited to chromosomal mutations and was basically non-transferable [44]. However, in 2015, findings from a study in China revealed the presence of a colistin resistance gene, mcr-1, in *E. coli* isolates from animals, food, and human bloodstream infections [46]. Colistin differs from third-generation cephalosporins in some critical One Health aspects of antibiotic resistance. These are associated with the accounts and style of colistin usage in humans and animals, as well as with the successive establishment of resistance to the polymyxin group of antibiotics, which were most likely triggered by the massive amounts of colistin used in animals rather than in humans [48]. In addition, the use of Avoparcin in animals has been linked to the choice and proliferation of vancomycin-resistant *Enterococcus* (VRE) species and glycopeptide-resistant genes in enterococci from animals, food, humans and the environment [49].

**Table 2:** Foodborne pathogens of human, animal and environmental significance.

Pathogen	Class of antibiotic Resistance	Transmission Route	Food Product Susceptible to Contamination	Reference
Nontyphoidal Salmonella *Campylobacter jejuni* *Escherichia coli* *Staphylococcus aureus*, Methicillin- resistant *Staphylococcus aureus* (MRSA) and other staphylococci *Listeria monocytogenes* and other *Listeria* species	Cephalosporin ^a,b^ Fluoroquinolone ^b^ Tetracycline ^b,c^ Penicillin ^a,b^ Sulfonamide ^b,c^ Fluoroquinolone ^b^ Macrolide ^a,b^ Cephalosporin ^a,b^ Fluoroquinolone ^b^ Carbapenem ^a^ Cephalosporin ^c^ Methicillin ^a,b^ Vancomycin ^a^ Cephalosporin ^a,b^ Penicillin ^a,b^ Fluoroquinolone ^b^ Tetracycline ^b,c^ Aminoglycoside ^a,b^ Carbapenem ^a^ Monobactam ^a^ Macrolide ^a,b^ Lincosamide ^c,d^	Faecal shedding into the environment Waste water, faeces and urine Water Contact with carrier animals; indiscriminate use of antibiotics in animals; negligence resulting in cross-infections within the confines of and amid farms; foreign trade in animal, food or supplementary outputs Sewage, effluent, faeces of man and animal, soil water	Meat and poultry products, fruits and vegetables Meat and poultry products Milk, meat and eggs Bacon, meat, milk and eggs Unpasteurized milk and its derivatives, meat, fish, chicken, poultry products, vegetables and salads	[38,40,50] [43,51] [40,52,53] [40,54] [55,56]

^a^ = critically important antibiotic to humans [57]. ^b^ = critically important antibiotic to animals [58]. ^c^ = critically important antibiotic to humans [57]. ^d^ = critically important antibiotic to animals [58].

## 4. Nanotechnology and One Health in Agriculture (Animal Husbandry)

The WHO has identified zoonoses as an area of research in which a One Health approach is particularly important. Zoonotic diseases are responsible for 75% of more recent human illnesses and are a huge global hazard to animal, human and food security [59]. Breakthroughs in science and technology have improved agriculture by providing fresh ideas and solutions to difficult challenges. Nanotechnology is constantly producing more effective and contaminant-free nano-formulations for sustainable agriculture [60].

On the basis of their significance in various sectors, NPs have drawn the attention of various research groups [61]. NPs have the prospect of transforming the agricultural sector by advancing management options with safer impacts on rampant infections in food-producing animals [60].

### 4.1. Synthesis of Nanoparticles

The typical absorption spectra of nanoparticles is below the critical wavelength of light, making them transparent [62]. They are able to travel through the vasculature and locate any target organ, which makes them incredibly beneficial for use in various industries, including the agriculture and medical sectors [63]. Based on the aforementioned, the creation of metallic NPs is a current field of academic and, more importantly, “application research” in nanotechnology. A multitude of procedures can be used to synthesis NPs. These methods are used to create dry particles as well as NPs in liquid dispersions. Building nanostructures from atoms (bottom-up) or shrinking the size of micro particles to NPs (top-down) are two methods for creating nanostructures [64].

#### 4.1.1. Top-Down Method (Physical Approach)

This method entails severing a mass of substances into nano-sized entities. The features of the NPs produced using this method are compromised because they lack appropriate surface structures [64]. Traditional physical processes, including spark discharge and pyrolysis, are used to create metal NPs [64]. Pyrolysis is the process of burning an organic precursor that has been pushed under intense pressure through an opening. These aforementioned “physical” methods for synthesizing metallic nanoparticles have a very low production rate and, more significantly, a very high cost [65]. Top-down production techniques result in product surface structure flaws, which is a significant drawback because the surface chemistry and other physical attributes of NPs depend heavily on the surface structure [66]. Wet-chemical techniques are utilized in the classic and most common ways of creating metallic nanoparticles. In a typical process, NPs are grown in a liquid medium that contains a variety of reactants, including reducing agents, such as sodium borohydride or potassium bitartrate. A stabilizing agent, such as polyvinyl pyrolidone or sodium dodecyl benzyl sulfate, is also added to the reaction mixture to prevent the agglomeration of metallic NPs [66].

#### 4.1.2. Bottom-Up (Chemical and Biological Approaches, Green Chemistry or Plant-Mediated Synthesis): An Approach Used for Synthesizing Plant-Derived Nanoparticles

Metal precursors, reducing agents and stabilizing/capping agents are the most common components used in the chemical approach. In general, there are two steps to the reduction of metal salts, namely, nucleation and subsequent growth.

Chemical procedures use water or organic solvents and other toxic materials, whereas green synthesis is a simple and convenient alternative to chemical and physical methods for the preparation of metallic NPs. Furthermore, ingredients utilized to make silver NPs (AgNPs), such as borohydride, thio-glycerol and 2-mercaptoethanol, are poisonous and dangerous [67]. Aside from the aforementioned drawbacks, the produced particles are not of the anticipated purity in that they exhibit chemical modifications on their surfaces. In addition, high radiation and extremely concentrated stabilizers and reducers, which are damaging to the environment and people’s health, have been used in chemical and physical procedures [68]. The production of NPs could be carried out using a wide variety of biological resources found in nature, including plants and plant products, algae, fungi, yeast, bacteria and viruses. It should be noted that inorganic compounds can be produced by both single-cell and multicellular organisms in the intra- or extra-cellular space [69]. In recent years, because of the great potential of plants to produce NPs of various shapes, and more importantly because of its eco-friendliness, green synthesis is the preferred method to employ in physical and chemical syntheses [70]. Green technology also allows for the one-step synthesis of NPs and requires less energy, which results in a variety of NPs with different natures, improved stability and suitable dimensions [68,71].

**Figure 2:**
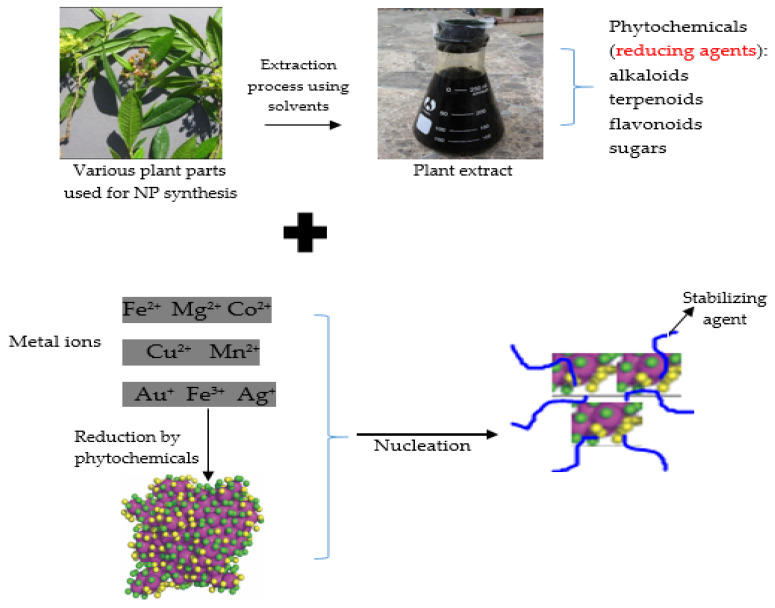
An outline of nanoparticles (NP) synthesis using plant extracts.

Green synthesized PDNPs also have some merits over antibiotics in terms of safety and activity in the human, animal and environmental health arenas (One Health) (Table 3). NPs are simply synthesized by mixing plant extracts with a solution of metal salts (Figure 2). Specific phases associated with the synthesis of plant-derived NPs include activation, growth and termination. Metal ions are reduced at the initial phase of activation and are followed by a growth phase, with the fusion of smaller NPs to form larger ones, and lastly, the termination stage, in which the ultimate size is achieved (Figure 2) [72,73]. As reducing agents, secondary metabolites are used by plants in the formation of NPs. In the process of producing NPs, biological agents are said to operate as reducers, stabilizers or both.

To attain a high level of availability and cheap manufacturing costs for these items, studies using indigenous/native plants should encompass multiple geographical locations [74]. The synthesis of metallic NPs using plant extracts has already been recorded [75,76,77]. Findings by Esmaeillou, et al. [78] revealed that silver NPs resolve vancomycin resistance in *S. aureus* by binding to the vancomycin and enhancing bacterial cell death. In addition, existing evidence indicates that the silver NPs are non-toxic [79,80]. However, evidence that NPs can be poisonous and harmful has been reported and this negatively influences living cells, especially at higher concentrations [81].

**Table 3:** Advantages of the green chemistry approach in the synthesis of safer and more sustainable nanoparticles from plant extract over antibiotics.

Green Synthesis of Plant-Derived Nanoparticles (PDNP)	Antibiotics	References
Efficient uptake of drug owing to their small sizes	Limited uptake of drug	[36,82]
Sufficient drug accumulation at target site	Reduced drug accumulation at target site owing to modification in target site	[83,84,85]
Pharmacokinetics: protection of encapsulated drug	Active drug efflux	[68,86]
Pharmacodynamics: retention of drug at active site increases bioavailability; thus therapeutic efficiency is enhanced and level of drug stability is increased	Inactivation of drug by cellular enzymes	[29,68]
Safety and activity: considerably safe and products have antibacterial properties	Resistance; a public health concern has developed on account of the indiscriminate use and the development and/or acquisition of resistant genes by pathogens	[24,87,88]
Minimal energy utilization, ecofriendliness, biocompatibility, and the use of renewable resources Cost-effective and easy to produce	Adoption of an organic chemistry method which uses chemicals, some of which may be dangerous and cause environmental concern Capital-intensive	[87,88,89] [70,90]

### 4.2. Characterization of Metallic Nanoparticles

NPs are characterized to evaluate their behaviour, bio-distribution, safety, efficacy and functional aspects. This is generally achieved by determining their size, shape, surface area, and level of dispersion. In order to assure reproducibility in their synthetic process, biological activity and safety, these NPs must be comprehensively and accurately described [91].

The characterization involves spectroscopic and morphological studies. For spectroscopic studies, analytical techniques are explored. Ultraviolet-visible (UV-vis) spectroscopy is a compulsory characterization technique that measures the optical properties of the NPs. With the help of UV-vis, an optical band gap can be calculated, which helps in classifying the materials for the purpose of energy conversion, such as light energy to electrical energy in solar cells [92]. In the case of ray diffraction techniques, X-ray diffraction identifies the crystal phase of the NP based on the position of a characteristic peak, while small-angle X-ray scattering (SAXS) detects the fractal structure of the NP agglomeration, determines its fractal dimension, finds the average radius of the agglomerates and primary particles, and is suitable for characterizing the structural characteristics of amorphous materials at relatively low resolutions [93]. X-ray photoelectron spectroscopy (XPS) is a valuable tool for studying the nature and consistency of NP surfaces. With proper sample cleaning, mounting, data collecting, and analysis, XPS can offer crucial quantitative information, including NP coatings, shells, and contamination [94]. Powder X-ray, electron or neutron diffraction is used to determine how NPs are arranged structurally. The amount of NPs in a unit as well as their size and distribution, affect a system’s performance or efficiency. Usually, concentrations are measured using a condensation particle counter (CPC) [95]. Furthermore, bulk solid phase samples are measured using laser diffraction techniques [96]. Using centrifugation and photon correlation spectroscopy, the particles in the liquid phase are measured. The purity and functionality of NPs are determined by their chemical or elemental make-up. Higher secondary or undesirable components may cause the NPs to be less effective, as well as cause secondary reactions and contamination during the process [97]. Fourier transform infrared spectroscopy (FTIR), is used as an in situ analysis of interfaces to investigate the surface adsorption of functional groups on NPs. It has a good signal-to-noise ratio, precision, and consistency. In order to conduct difference spectroscopy, one needs to be able to detect minor absorbance variations on the order of 10^−3^, which makes it feasible to separate the small absorption bands of functionally active residues from the massive background absorption of the total protein [98]. Nuclear magnetic resonance (NMR) reflects the dispersion and compatibility of nano-emulsions in water, as well as the state of each component molecule in the colloidal system [93]. A potent method for surface analysis is surface-enhanced Raman spectroscopy (SERS), whose probes have the narrowest emission peaks and the highest multiplexing capacities [99]. SERS probes also have the benefit of withstanding harsh environmental conditions (such as variations in humidity, pH and ionic strength) while still producing a powerful emission signal [100]. Particularly, a long-standing restriction is its lack of content and morphological generality. However, the development of shell-isolated nanoparticle-enhanced Raman spectroscopy (SHINERS) solves this issue [101]. On the other hand, the morphological properties of NPs are measured by using dynamic light scattering (DLS) and an electron microscopy. One of the most fundamental and significant measurements for characterizing NPs is particle size. DLS is the most common approach to analyse the hydrodynamic particle size and the distribution of the particles over a range of sizes. DLS measures light interference based on the Brownian motion of the NPs [102]. Electron microscopy can be employed for revealing the details of the NP shape and surface and such techniques include: scanning electron microscopy (SEM), which determines the size distributions, shapes and surface morphology; and transmission electron microscopy (TEM), which quantitatively measures the particle size, distribution and morphology [103,104,105]. However, poor quality electron microscopy pictures are produced by particles covered with biomolecules [102].

## 5. Applications of Plant-Derived Nanoparticles in the Food Industry

NPs can be applied in enormous fields, including food industry. Processing, storage and packaging operations are only a few of their respective uses in the food industry. Owing to the greater surface area of NPs per unit of mass, it is to be expected that as opposed to the macro-sized particles of the same chemical make-up, they would be more active biologically and thus offer various approaches in respect to food applications [17]. The use of nanotechnology as an alternative to antibiotics in the treatment of infections in food-producing animals has gained significant recognition in recent times (Table 4) [106]. In addition, incorporating nanotechnology into food manufacturing, processing, protection and packaging improves the quality of the product [107]. In the case of food packaging, a nanocomposite coating can directly incorporate antimicrobial chemicals onto the coated film surface [108]. One example is the canola oil manufacturing sector, which uses nano drops, a food ingredient meant to transmit vitamins and minerals [95].

Several studies have also reported the activity of PDNPs against antimicrobial-resistant pathogens. Sani, et al. [109] reported the activity of silver (Ag) and copper-oxide (CuO) NPs synthesized from the aqueous leaf extract of *Carica papaya*. Another study reported the minimum inhibitory concentration (MIC) and minimum bactericidal concentration (MBC) of *Terminalia catappa* leaf extract (TCE) synthesized AgNPs (TCE-AgNPs) against multidrug-resistant *P. aeruginosa* (MDR-PA) as 3.88 ± 0.13 and 7.77 ± 0.25 µg/mL, respectively, and 7.77 ± 0.25 and 31.08 ± 1.01 µg/mL against methicillin-resistant *S. aureus* (MRSA), respectively [110]. These studies concluded that the produced PDNPs can be explored as substitutes for addressing AMR in the examined MDR bacterial strains.

**Table 4:** Antibacterial activity of plant-derived nanoparticles. NM = not mentioned.

Plant Used	Plant Part Used for Extraction	Solvent Used for Extraction	Phytochemicals	Nano-Particle	Target Pathogen	Reference
*Aegle marmelos*	Fruit	Methanol	Tannins, saponins, steroids, alkaloids, flavonoids, glycosides	Ag	*Bacillus cereus*, *Pseudomonas aeruginosa*, *Salmonella dysentriae*	[111]
*Allium rotundum*	Leaf	Deionised water, ethanol	Terpenes, phenol, carvacrol	Ag	*Pseudomonas aeruginosa*, *S. aureus*	[112]
*Aloe vera* and *Linum usitatissimum*	Leaf and seed	Distilled and deionised water	Phenolics, phenolic glycosides	Fe_2_O_3_	*S. aureus*, *Salmonella typhi*	[113,114]
						
*Annona muricata*	Leaf	Deionised water	Flavonoids, terpenoids	Au	*S. aureus*, *Entrococcus faecalis*, *Klebsiella pneumonia*, *Clostridium sporogenes*	[115]
*Ashwagandha, bufera*	Leaf	Water	Flavonoid, tannin	Se	*Bacillus subtilis*	[116]
*Asparagus racemosus*	Root	NM	Phenols, tannins, sterols	Pd	*S. aureus*, *E. coli*	[117,118]
						
*Caesalpinia bonducella*	Seed	NM	Citrulline, phytosterinin, flavonoids	CuO	*S. aureus*, *Aeromonas* species	[119]
*Camellia sinensis*	Leaf	Water	Polyphenol	NiO	*S. epidermidis*, *Pseudomonas aeruginosa*	[120]
*Catharanthus roseus*	Leaf	Water	NM	Ag	*Shigella dysenteriae*, *Klebsiella pneumoniae*, *Bacillus anthraces*, *Staphylococcus aureus*, *Pseudomonas aeruginosa*	[121]
*Chromolaena odorata* *Clerodendrum inerme*	Root Leaf	Coconut sap Fruit juice	Alkaloid Terpenoids, tannins, saponins, alkaloids, phenolics, cardiac glycosides, anthraquinones	Fe_3_O_4_ Ag, Au	*E. coli*, *S. aureus* *S. aureus*, *B. subtilis*, *E. coli*, *Klebsiella* species	[122,123] [124]
*Cocos nucifera*	Inflorescence sap	Methanol, chloroform, water	Flavonoids	Ag	*Bacillus pumilus*	[125]
						
*Datura metel*	Leaf	Water	Alkaloid, flavonoid	CeO_2_	*Enterococcus faecalis*, *S. aureus*, *Klebsiella pneumonia*, *E. coli*	[126,127]
*Diospyros kaki*	Peel	Methanol	tannins, carotenoids, flavonoids, steroids, lipid, terpenoids, naphthoquinones	MgO	*S. aureus*, *E. coli*	[128]
*Euphorbia* *heterophylla*	Leaf	Water	Alkaloid, flavonoid, saponin, tannin	MnO_2_	*E. coli*, *S. aureus*, *Streptococcus mutans*	[129,130]
*Galphimia glauca*	Leaf	Water	Tri-terpenes, galic acids, terpenoids, phenolics	Ag	*Pseudomonas aeruginosa*	[131]
*Gardenia jasminoides*	Leaf	Water	Polyphenol, flavonoid	Cu	*S. aureus*, *E. coli*	[132]
*Leucaena leucocephala*	Leaf	Water	Flavonoids, coumarins, tannin, saponin, phenol, steroid, Cardial glycoside	CdO	*Pseudomonas aeruginosa*	[133]
*Musa paradisiaca*	Stem	Water	Glycosides, flavonoids and terpenoids	Ag	*Bacillus subtilis*, *E. coli*	[134]
*Tamarix nilotica*	Shoot	Water	Phenol	Ag	*Listeria monocytogenes*	[135]
						
*Trigonella foenum-graecum*	Leaf	Water	NM	TiO_2_	*Bacillus subtilis*	[136]

### 5.1. Function and Significance of Natural Products of Plants in the Activity of Plant-Derived Nanoparticles

The plant kingdom generates a large range of metabolites with far-reaching biological and pharmacological effects. There are approximately 200,000 identified phytochemicals among the 300,000 plants on our planet [137,138]. Plants use primary metabolites, including carbohydrates, fatty acids, nucleic acids and amino acids, as well as other components, to grow, while secondary metabolites are produced in response to a variety of biotic and abiotic stresses [139,140]. Additionally, plant-produced phytochemicals, such as polysaccharides, polyphenolic alkaloids, saponins and terpenoids, reduce metal ions or metal oxides into zero-valence metal NPs (Figure 2) [141]. As a result, many functional groups (e.g., free carboxylic, alkenyl, amide, amine, phenolic and alcohol groups) found in plant extracts are primarily engaged in biological reduction and bio-capping. Hard ligands have at least two polyhydroxyl (–OH) groups at para/ortho positions and engage in soft metal reduction, whereas carboxylic (–C=O) groups operate as soft ligands and contribute to surface capping by generating an electrostatic connection with soft metals [142].

The capacity of plant secondary metabolites to bind to or conjugate with NPs upon green synthesis may be used for the purification of chemicals for drug discovery [143]. Proteins, sugars, terpenoids, polyphenols, alkaloids and phenolic acids aid in the reduction of metal ions into NPs and maintain their stability afterward [144]. Different solvent extracts differ in the concentration of molecules that serve as reducing and stabilizing agents for the creation of NPs [145].

The biomolecules with carboxyl, amine and hydroxyl functional groups were engaged in the reduction of Au ions, according to an analysis of Au NPs that were green-synthesised utilizing *Suaeda monoica* leaf extract [146]. The green synthesis of NPs was accomplished with the use of isolated flavonoids and terpenoids. The ability of the terpenoid fractions from *Andrographis paniculata* leaves to produce ZnO NPs via green synthesis is further demonstrated by the presence of the C=O functional group in the NPs [147].

### 5.2. Antibacterial Effects of Plant-Derived Nanoparticles (PDNPs)

PDNPs eradicate bacteria to heal diseases by employing various mechanisms whereby they interact with the bacterial cell wall and cell membrane and thus alter the metabolic activity of the cell. Drug-delivery systems consisting of NPs offer a variety of functional and biological features [148,149]. They are readily adjusted in that they modify the dosage and ratio of the constituents of the drug, as well as the components used in their manufacture, to address issues that are associated with conventional antibiotics [150]. In recent years, studies have been conducted on the enhanced antibacterial activity of NPs against resistant *S. aureus* isolated from bovine mastitis [151,152]. These NPs could include one or more medications without damaging the structure of the material, hence enhancing the pharmacological effectiveness of the agent [153]. They offer several advantages, including a consistent form of medication dosage, increased bioavailability, the delivery of the medicine to the infected site, reduced therapeutic time and adverse effects, and the prevention of burst release and drug degradation. Generally, they are safe for public health and the environment [150].

In addition to their vital role in protecting drugs from degradation and delivering them to diseased sites, nanomaterials can be cytotoxic and damaging to bacteria [152]. Although in-vitro toxicity testing does not guarantee the same outcomes as in vivo testing, it provides an indication regarding toxicity. This justifies and serves as motivation for in vivo studies from economic and ethical perspectives. In-vivo studies are reliable, more informative and good predictors of long-term physiological effects. Evaluations of in-vivo toxicity remain the gold standard for determining how poisonous NPs are [154]. Particularly, NPs interact with the bacterial cell membrane, causing it to break down, thus releasing reactive oxygen species (ROS), and causing enzyme inactivity, protein deactivation and altered gene expressions, as well as stimulating specific and non-specific immunity [155]. Such antibacterial mechanisms aid NPs in their fight against antibiotic resistance. In the synthesis of zinc oxide (ZnO), for example, phytochemicals present in the *Bauhinia tomentosa* leaf extract exerts a bio-reducing property. Thus, the resulting ZnO NPs could be used as a potent antibacterial force in biological applications [156].

### 5.3. The Inhibition of Biofilm Formation by Plant-Derived Nanoparticles (PDNPs)

According to Chakraborty et al. [157], biofilms are compounded networks of bacterial populations which form an attached, localized microenvironment that is protected by an exopolysaccharide extracellular matrix. The ability of PDNPs to infiltrate biofilms suggests a feasible method for preventing biofilm development [158]. The glycocalyx, which has an anionic charge, is the most important component of the biofilm; it may interact with NPs that have a positive charge, thus permeating the thick biofilm [159]. Many studies have been conducted on the activity of PDNPs against bacterial pathogens. For instance, gold (Au) NPs caused significant reduction of biofilm structure formed by *S. aureus* [160]. In another study, triclosan revealed bactericidal properties to *S. aureus* outside of the biofilm, but when combined with a micellar nano carrier, it was able to probe staphylococcal biofilms and kill all the bacteria cells around the biofilm [161].

Some studies have demonstrated that interference with the quorum-sensing systems (QSs) of microbes can serve as a key regulatory mechanism in biofilm growth, thus preventing the formation of biofilm [162]. In a multi-drug-resistant *E. coli* strain obtained from a dairy cow with mastitis, QA NPs, a composite material containing AgNPs, and the plant-derived therapeutic component, quercetin (Qe), outperformed AgNPs and Qe used independently in terms of their antibacterial and anti-biofilm capabilities [163]. Additionally, Zn^2+^ ions may be used as an efficient antibacterial treatment in a variety of dairy applications, including biofilms and vegetative bacterial cells [164].

### 5.4. Parameters Affecting the Antibacterial Activity of Plant-Derived Nanoparticle

Temperature, pH, surface charge, reaction time and the ratio of biological extract to inorganic compound (metal salt) are parameters that must be addressed while synthesizing metal NPs. The entire output of the NP is influenced by several characteristics [165]. According to findings, changing the pH induces variations in the zeta potential of NPs because the cationic nature of the metallic ion changes as the ionic strength of the solution varies [166]. Similarly, raising the reaction temperature increases the reaction rate, which affects the heat stability of the reducing chemical and, as a result, the yields. In most circumstances, time, pH and temperature are proportional to the rate of response. Finally, the amount of plant extract and metal salt in the combination determines the size and form of the NP [167,168].

Antibacterial activity was shown to be stronger in smaller particles [169,170] and vice versa [171]. NPs with a smaller ratio of surface to volume present with an increased level of concentration of metal molecules, thus improving the interaction of such NPs with the pathogen cell walls/membranes and again boosting the generation of ROS. Smaller particles are also more likely to enter microbial cells, where they interact with their intracellular features [172]. According to Oliver, et al. [173], antibacterial actions of AgNPs larger than 20 nm rely on the discharge of Ag+ ions, but those smaller than 10 nm are more potent antimicrobials because they penetrate microbial cells directly and interact with their biological elements and enzymes. The antibacterial effectiveness of AgNPs is likewise highly correlated with their concentration. The higher the NP concentration, the stronger the antibacterial action, and vice versa [174,175]. MICs of AgNPs are at odds with bacterial food pathogens, such as *Lactobacillus acidophilus* and *Lactobacillus fermentum*, and have recently varied from 15 to 90 mg/mL [176]. The biofilms of *S. aureus* and *P. aeruginosa* isolated from food were found to be susceptible to a concentration of 62.5 mg/mL of AgNP, whereas doses of 125 and 250 mg/mL of the same nanoparticle inhibited biofilms by 85 and 90%, respectively [177].

Also, the form of a NP is related to how it interacts with the cell wall/membrane of a microorganism. Antibacterial characteristics of metal NPs in various shapes and sizes, such as triangular and oval, and of crystalline composition, have all been studied [178,179]. Acharya, et al. [180] reported that as opposed to rod-shaped and spherical NPs, triangular-shaped AgNPs were found to present with better microbiological activity against *E. coli*. However, it is noteworthy that the data on the influence of AgNP shape on antibacterial effectiveness are disputed, as there are studies contradicting the former [181].

## 6. Conclusions and Future Prospect

Plant extracts are often safe and eco-friendly to synthesize NPs. One of the most notable advances in the green synthesis of metallic biogenic NPs might in fact be a beneficial technique for determining NPs’ mechanism of action. This is a carefully regulated synthesis that is simple to scale up and provides the assurance of a safer space and sustainability. Plant-derived NPs have demonstrated a variety of benefits and uses in the food sector. Particularly, studies show that NPs can be antibacterial, thus ameliorating the current problem of acquired resistance caused by the abuse or overuse of antibiotics.

Considering this possible benefit, future research should concentrate on determining the cytotoxicity of plant-based NPs. Additionally, in vitro toxicity studies cannot be generalized to in vivo levels. Hence, thorough in vivo toxicity (bio-compatibility) investigations of plant-derived metal NPs in animal models remain essential following the positive effects that have been demonstrated under in vitro conditions. It is imperative to understand the antibacterial effects of NPs in the body systems of both humans and animals, which is linked to PDNPs’ pharmacodynamics. To transform PDNPs into a feasible approach capable of meeting society’s desire for an effective remedy against antibiotic resistance, more research on pharmacodynamics is required, as is an investigation of the mechanisms of action that mediate the antibacterial impact of NPs.
